# Behavioural intentions in response to an influenza pandemic

**DOI:** 10.1186/1471-2458-10-174

**Published:** 2010-03-30

**Authors:** Gerjo Kok, Ruud Jonkers, Roger Gelissen, Ree Meertens, Herman Schaalma, Onno de Zwart

**Affiliations:** 1 Work and Social Psychology, Maastricht University, PO Box 616, 6200 MD Maastricht, the Netherlands; 2 ResCon Research & Consultancy, Rijswijkstraat 175, 1062 EV Amsterdam, the Netherlands; 3 Health Promotion, Maastricht University, PO Box 616, 6200 MD Maastricht, the Netherlands; 4 Municipal Public Health Service Rotterdam-Rijnmond, Schiedamsedijk 95, 3011 EN Rotterdam, the Netherlands

## Abstract

**Background:**

Little is known regarding which behavioural responses can be expected if an influenza pandemic were to occur.

**Methods:**

A survey comprising questions based on risk perception theories, in particular PMT, was conducted with a Dutch sample.

**Results:**

Although fear that an influenza pandemic may occur was high, participants do not feel well informed. General practitioners and local health authorities were considered trustworthy sources of information and the information considered most urgent pertained to which protective measures should be taken. Participants reported an intention to comply with recommendations regarding protective measures. However, response and self efficacy were low. Maladaptive behaviours can be expected. Increasing numbers of ill individuals and school closures are also expected to lead to a decreased work force. Participants indicated wanting antiviral drugs even if the supply were to be insufficient.

**Conclusions:**

Messages regarding health protective behaviours from local health authorities should anticipate the balance between overreacting and underreacting. Also, when protective recommendations from health professionals conflict with company policies, it is unclear how employees will react.

## Background

Currently, both the World Health Organization and the European Union urge countries to prepare for a possible influenza pandemic [1], as Influenza A(H1N1) or, alternatively termed, the Mexican swine flu has the potential to develop into a pandemic. A pandemic is likely to occur when a new and severe type of influenza virus for which humans are not immune evolves. A pandemic is a worldwide epidemic that can cause significant morbidity and mortality. People's behavioural responses can impact the societal consequences of a pandemic. However, to date, we know little about how people are likely to react to a pandemic crisis. It is consequently difficult to develop effective communication strategies and behavioural interventions that anticipate people's possible reactions and subsequently limit the consequences of a pandemic if it were to occur. Given this deficit of knowledge, this study investigated possible behavioural reactions to an influenza pandemic among people living in the Netherlands.

### Theoretical background

The literature on behavioural responses to an influenza pandemic refers frequently to a number of theories on risk perception [2-5] including the Protection Motivation Theory (PMT) [6], the Health Belief Model (HBM) [7], the Extended Parallel Process Model (EPPM) [8] and the Precaution Adoption Process Model (PAPM) [9]. The basic idea underlying these theories is that people react to a threat by performing some action. The PMT distinguishes two phases, namely a threat appraisal and a coping appraisal. The threat appraisal is determined by perceived personal susceptibility (or perceived vulnerability) to the threat (beliefs about the likelihood of getting a disease or condition) and perceived severity of the threat (feelings about the seriousness of contracting an illness) which combine to generate fear arousal. The coping appraisal is determined by variables relating to the suggested protective response. These are perceived response efficacy ("What protective behaviour will help?"), perceived self efficacy for the response ("Am I confident that I can perform the protective behaviour?") and the response costs ("What are the disadvantages of the protective behaviour?"). According to the PMT, the threat appraisal stimulates the intention to act while the coping appraisal determines the type of action employed. The action may be adaptive and lead to the performance of the advised protective or precautionary behaviours or it can be maladaptive thus leading to the performance of behaviours other than those advised. In order to act adaptively, perceived severity and susceptibility (i.e. the threat) as well as response and self efficacy must be high while response costs remain relatively low. If perceived severity and susceptibility are low, people will not perceive the matter as threatening and will consequently not be inclined to act. If perceived severity and susceptibility are high but response and/or self efficacy are low, maladaptive responses (e.g. denying the existence of a threat) are likely to ensue[10,11].

### Empirical evidence

To date, a limited number of studies that focus on behavioural responses to an influenza pandemic are available. One is a study conducted by Markel and colleagues [12]. In their study, they described the effects of a number of nonpharmaceutical interventions that were implemented in US cities during the 1918 and 1919 influenza pandemic. These included school closures, bans on gathering in public places, isolation and quarantine, and ancillary interventions, such as work schedule alterations, transportation restrictions, and face mask ordinances. Their findings suggested that the application of these behavioural interventions did indeed limit the consequences of that pandemic.

Following the severe acute respiratory syndrome (SARS) epidemic in 2003, Sadique and colleagues [4] conducted a population-based survey on people's protective actions in response to a hypothetical influenza pandemic in five European and three Asian regions. With few exceptions, the patterns of potential protective actions were similar among participants in each region. Public transportation was generally regarded as the most risky site for infection, while one's home was seen as the least risky setting. Participants indicated that if a new influenza pandemic were to occur, they would likely limit their use of public transportation and avoid places of entertainment and shopping for nonessentials. Participants also reported considering their risk for infection high in health care facilities but did not indicate that they would avoid such facilities. Further, employed participants were less likely to report that they would take protective actions. Interestingly, risk perception variables did not substantially impact protective behaviours. The only exception pertained to the avoidance of public transportation.

Hong and Collins [13] examined risk perceptions and protective behaviour in Korea. They reported that Korean public health agencies have promoted influenza vaccinations as a protective measure against SARS, even though the effectiveness of influenza vaccinations in reducing SARS incidence is doubtful. The results of this study showed that both enhanced risk perceptions and the belief that influenza vaccinations reduce the threat of SARS increased influenza vaccination intentions. In essence, this study demonstrated that participants adopted recommended protective measures even when their effectiveness was questionable.

De Zwart and colleagues [3,14,15] studied people's responses to recent outbreaks of SARS and Avian Influenza (AI). They found that not only risk perception but also communication variables such as information quality, communication preferences, trust in the source of information, and perceived urgency of the information impacted people's responses. In one study, protective behaviours against the 2003 SARS outbreak were investigated [15]. The results indicated that few people took protective measures such as wearing face masks, washing their hands more frequently, endeavouring to get extra sleep, consulting a doctor when potential symptoms present and paying more attention to coughing. All of the above measures were reported in less than 10% of the sample and only small differences between the Dutch and the Finnish samples were found.

In a meta-analysis of 34 studies, of which 25 focused on influenza, Brewer and colleagues [2] investigated the relationship between risk perception and vaccination behaviour in adults. The results showed that perceived susceptibility and perceived severity were both positively related to actual vaccination behaviour. However, experimental studies were recommended in order to determine the direction of these relationships.

Some authors have explored people's responses to disasters and other crises, including pandemics. Fischhoff [16] conducted a survey study among American citizens that investigated expected responses to possible health threats (i.e. anthrax, West Nile virus, smallpox and the dirty bomb) on the part of the public and the public authorities. They concluded that people were poorly informed about most of these threats and did not know which responses would be most appropriate. Inappropriate responses such as fleeing were thus considered probable by the study participants. However, according to Fischoff and colleagues, people rarely actually panic unless they have lost faith in public authorities. Nonetheless, many of the study participants conveyed an expectation that other people would panic and thus supported compulsory treatment and isolation.

Crimando [17] developed a guide for disaster preparedness "based on a thorough review of current research and literature, expert consensus and field experience in disaster and terrorism response and planning" (p. 1), and claimed that most people respond to disasters in one of three ways The first and most common behavioural response is neighbor-helps-neighbor. When this happens, people help each other and follow instructions. The second response is neighbor-fears-neighbor. This is when others around us are perceived as part of the problem, and can be the case when the threat is an infectious disease. The third and statistically most unlikely response is neighbor-competes-with-neighbor. This is essentially panic and is considered to be the consequence of perceptions that opportunities to escape and the availability of critical supplies are limited. Crimando further claimed that, although people's safety is the first concern in a disaster, effort is necessary to ensure that businesses and companies survive the disaster so that people will still have employment when the disaster is over.

### Research question

The objective of this study was to determine how people intend to behave when a large scale influenza pandemic occurs. The results provide relevant information that furthers our theoretical understanding of risk behaviour intentions and enables us to develop better and more effective policy responses to future pandemics.

## Methods

### Procedure

A survey based on PMT and complemented with relevant concepts suggested in the literature was conducted with a Dutch sample. Participants were asked how they would act during a pandemic depending on the severity of pandemic, the protective instructions provided and the communication strategies employed. It should be noted that the questions on these concepts are meant to give insight into how respondents perceive the threat, rather than their knowledge of the facts on this issue, as behaviour is determined by how people perceive their environment to be, instead of by the objective facts. From a panel of online research participants aged 18 and older, 1099 participated in the study (response 62%). Participants received a small monetary reward.

### Measures

#### Demographics

Household constitution was determined with items on the number of family members and number of children under 12 and under 18 residing in the household.

#### Information

Knowledge was measured as follows: First, participants were asked if they were familiar with the concept influenza pandemic. Answers were provided on a 3-point scale with the answer options 'Yes, I know what it is', 'I have heard of it but I am not sure what it is' and 'No, I have never heard of it'. Then the following text was provided: *"A pandemic is a worldwide epidemic caused by a virus that is as yet unknown and for which no vaccine is available. An example is the Asian bird flu. When that virus infects people, a new and unknown form of influenza that is transmitted from person to person develops. It is not possible to predict whether or not a new influenza virus is in fact dangerous and will cause an epidemic or a pandemic." *Following the above text, a second familiarity question similar to the one used prior to the text was posed. Estimation of the consequences was measured with an item asking participants how many people they think would get sick if a pandemic were to occur. Being informed was measured with items that asked participants the extent to which they think they are well informed about influenza viruses and epidemics, and about protective actions. These items were answered on a 8-point scale ranging from 'incredibly well informed' to 'very poorly informed'. For the analyses, these answers were collapsed to generate three categories, namely 'well informed', 'moderately informed' and 'poorly informed'.

#### Threat appraisal

Perceived susceptibility was measured with the item "How likely is it that you will get influenza from a new influenza pandemic in the next 12 months?" and perceived severity was measured with the item "How awful would it be if, in the next 12 months, you got influenza from a new influenza virus that has become a worldwide pandemic?". These items were scored on a 10-point scale with a higher score indicating greater perceived susceptibility or severity. Both items were also used to appraise nine other diseases. Fear was subsequently measured with the item "Are you scared a worldwide influenza pandemic will occur?" Answers were provided on a 5-point scale ranging from 'constantly' to 'never'. The perceived risk of infection in eight possible public places was measured with the item "In which of these places do you think you are most likely to get infected?"

#### Coping appraisal

Response efficacy was measured with the item "Can people take protective measures?" and self efficacy was measured with the item "Are you able to take protective measures?" Both were scored on a 5 point scale that ranged from 'yes, I am very certain that the measures could be taken/that I could take the measures' to 'no, I am certain that the measures could NOT be taken/that I could NOT take the measures'. To measure response costs, the following scenario and question was presented to the participants: *"Imagine, there is a new worldwide influenza pandemic that has also reached the Netherlands. The local community health services have provided you with a face mask and urged you to wear it in all public places in order to prevent infection. Would you wear the face mask in all public places?" *Those participants that indicated a willingness to wear the face mask were provided with an additional scenario in which the response costs were increased. These included *"people may think you have the new influenza and avoid you" *and *"the face mask causes skin irritation"*. After reading the scenario with the additional response costs, participants were asked again if they would wear the face mask in all public places. Answers were provided on a 5 point scale ranging from 'certainly' to 'certainly not' with an 'I don't know' option.

#### Behavioural responses

Maladaptive behavioural responses were measured by asking participants to react to ten statements, each of which represented one of the following maladaptive responses: avoidance, denial, fatalism, wishful thinking, and despair [18] (see Results) These were scored on a 5 point scale ranging from 'totally agree' to 'totally disagree'. Adaptive behavioural responses were measured by first presenting the following hypothetical scenario: *"Imagine a new worldwide influenza pandemic occurs and this pandemic reaches the Netherlands. Within five weeks, 400,000 Dutch will become ill and 4,000 people will die."*, and then indicating that health professionals recommend taking certain protective measures. For nine different instructions for protective measures, participants were asked, *"For how long would you be willing to take this measure?" *(see Results). Answers options were 'more than a month', 'a few weeks', 'a few days', 'I am not willing', and 'I don't know'.

#### Communication

Perceived trustworthiness of information sources was measured by asking participants to indicate first the degree to which they consider ten sources of information to be trustworthy sources of information in general and then the degree to which they consider these sources to be trustworthy sources of information if a worldwide influenza pandemic were to occur (see Results). Urgency of information was measured by asking participants to select two of seven topics they would like to receive information about immediately (see Results).

#### Scenarios

Participants were posed a series of possible scenarios that could take place if a pandemic were to occur. These were the participant becomes ill, a family member becomes ill, the participant becomes less able to work, the schools close, and antiviral drug availability is limited. In the scenario pertaining to the participant falling ill, participants were asked if they would stay home and if someone would be able to take care of them. In the scenario pertaining to a family member falling ill, participants were asked if they would stay home and take care of that person. If participants answered affirmatively, they were asked if they could continue to work. In the scenario that followed, participants were asked to imagine that the government has asked all people with influenza to stay home, that they have influenza symptoms, and that their employer nonetheless demands they come in to work. Participants that reported being willing to stay home despite their employer's demand were then confronted with a scenario in which the consequence of staying home would be a loss of income. Another scenario described a situation in which companies were advised to close their doors and subsequently did not pay their employees. For the previous two scenarios, participants were asked how likely that scenario would be and how long it would take before their household would encounter serious financial difficulties. Participants with children younger than 12 were also given a scenario in which all schools and child care facilities were closed for a month. Participants were asked if they would be in a position to care for their children themselves or if they could arrange alternative child care arrangements. Participants with children younger than 18 were also given the following scenario: *"Imagine all schools and child care facilities close for three months in order to protect children from influenza infection. Health professionals have advised you to keep your child(ren) away from public places such as shopping centres and public transportation for three months. Children should also discontinue social contact with other children. Would you be able to keep your child from doing these things for three months?" *The final scenario pertained to antiviral drugs and was as follows: *"A vaccine for a new influenza virus can only be developed after the outbreak. There are, however, antiviral drugs such as Tamiflu and Relenza that may prevent the virus from dispersing through the body and that can shorten the illness period. However, it is unclear whether these antiviral drugs actually work for the new influenza infection." *After having read the scenario, participants were asked if they would want to use the drug and if they would be agree with a policy that prioritizes the treatment of vulnerable groups, such as the elderly and professionals such as the police and fire fighters. If so, they were asked if they would attempt to obtain access to the drugs via other means.

## Results

### Participants

Table 1 displays demographic characteristics of the sample (n = 1099). The sample comprised slightly more women, elderly people and people with a higher level of education than the average Dutch population over the age of 18.

**Table 1:** Sample characteristics *(n *= 1099)

Characteristics	Sample percentage	Population ≥ 18 percentage
Gender		
Male	48	50
Female	52	50
Age		
18-29	15	19
30-39	17	18
40-49	20	20
50-59	18	18
> 60	30	25
Education		
Low	32	33
Moderate	36	41
High	32	25

### Knowledge about an influenza pandemic

Prior to the provision of a definition, 46% of participants were familiar with the concept of an influenza pandemic, 27% indicated being unsure and 27% were unfamiliar with the concept. After the provision of the definition and information on an influenza pandemic, 21% reported having been completely familiar with the information, 38% were mostly familiar, 18% were somewhat familiar, 12% were vaguely familiar and 12% were not at all familiar with the information. Although authorities estimate that 30% people in the Netherlands would get sick if an influenza pandemic were to occur, participants in this study estimated that number at 35% with a great deal of variation (sd = 22). When asked whether they perceived themselves to be informed about influenza viruses and pandemics, 26% considered themselves to be well informed, 63% claimed that they were moderately informed and 12% poorly informed. Regarding preventive actions, 18% considered themselves well informed, 53% claimed to be moderately informed, and 29% considered themselves poorly informed.

### Threat appraisals

**Figure 1:**
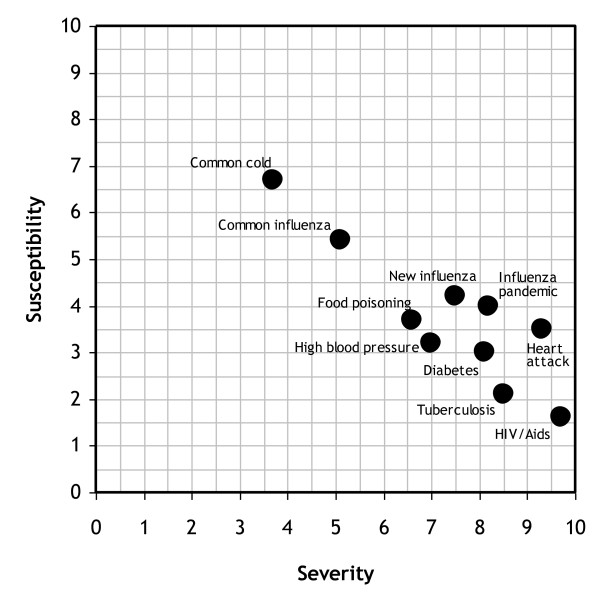
**Severity and susceptibility of an influenza pandemic compared to other diseases (the dots represent mean responses)**.

Regarding the perceived susceptibility of acquiring influenza in the coming 12 months and the perceived severity of that infection, a negative relationship was found between severity and susceptibility. Comparisons across medical conditions (see Figure 1) showed that HIV was considered the most severe but least likely disease while a common cold was considered least severe but most likely. A common influenza was thought to be somewhat more severe but less likely than a common cold. However, when the influenza was thought to be caused by a new virus, it was considered more severe than a common influenza. Susceptibility did not differ significantly between a common influenza and an influenza caused by a new virus. When influenza was considered pandemic, participants reported higher levels of perceived severity. Only tuberculosis, a heart attack and HIV were thought to be more severe. One way to conceptualize risk perception is to consider it the product of perceived severity and perceived susceptibility [19]. This computation yielded HIV as the least risky disease and an influenza pandemic as the most risky disease.

Regarding fear, only 5% of participants indicated being constantly scared that an influenza pandemic will occur while 35% reported being afraid sometimes, 44% rarely and 15% never.

An analysis of the intercorrelations between the three threat appraisal variables (perceived susceptibility, perceived severity and fear) demonstrated relatively low coefficients (between .10 and .27) thus suggesting that the four variables are indeed distinct aspects of threat appraisal.

Regarding risky locations for acquiring an influenza infection, 24% of participants claimed that public transportation poses the greatest risk, 20% reported that public places such as restaurants, bars and theatres are most risky, 17% claimed that places of employment and schools were most risky, 14% considered the most risky location to be shops and 11% claimed that hospitals pose the greatest risk.

### Coping appraisals

With respect to response efficacy, 5% reported being certain that people would be able to take protective measures if need be, 16% reported being quite certain, 42% were somewhat certain, 28% were doubtful and 9% were absolutely certain that they could not take the necessary protective measures. Regarding self efficacy, 5% reported being certain that they would be able to take the necessary measures to prevent infection, 17% were quite certain, 39% were somewhat certain, 29% were doubtful and 11% reported that they would not be able to take the measures necessary to prevent infection. Interestingly, the distributions for these two items were almost the same (*r *= .71). Participants' expectations were also relatively negative. With respect to behavioural costs and in response to the initial scenario posing relatively few costs, 35% of the participants reported being completely willing to wear face mask in all public places, 44% claimed that they would probably be willing, 13% indicated that they would probably not be willing, 2% were certain that they would not wear a face mask and 6% were unsure. Among the 79% that indicated that they would be (probably) willing to wear a face mask in all public places, 29% indicated to they would certainly wear a face mask even if it was stigmatizing or irritating, 56% indicated probably, 10% probably not, 1% certainly not and 5% indicated that they were unsure. All in all, approximately one third of the participants reported that they would probably wear a face mask even if it is stigmatizing or irritating.

### Maladaptive responses

**Figure 2:**
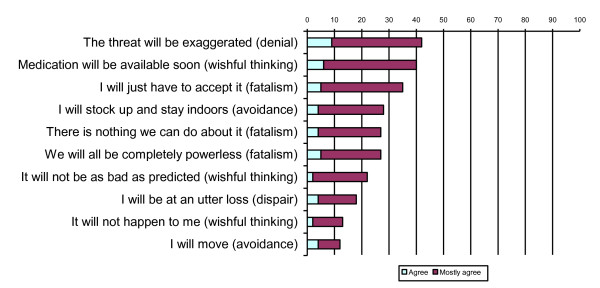
**Maladaptive behavioural responses to an influenza pandemic**.

Approximately 40% of all participants expected that the government and/or media would exaggerate the severity of an influenza epidemic, that medication for the virus would become available soon and that the pandemic simply has to be accepted as reality. Few participants reported an intention to flee. Only 4% agreed completely with the statement and 8% agreed mostly. Despair was reported by 18% while stocking up and staying indoors was reported by 28% (see Figure 2).

To confirm the theoretical distinction between the five subscales, a principal components analysis with varimax rotation was applied. The ten statements can be summarized in three factors with eigenvalue > 1, (see Table 2). Based on factor loadings > .30, the first factor can be interpreted as fatalism, the second as underestimation combining denial and wishful thinking, and the third as avoidance. The item representing despair contributes to both fatalism and avoidance.

**Table 2:** Factor analysis of maladaptive responses (*n *= 1099)

Statements	Fatalism	Underestimation	Avoidance
We'll move to a place without influenza			.81
We should stock up and stay indoors			.81
I'm at an utter loss	.35		.58
We are all completely powerless	.85		
There's nothing we can do	.89		
We just have to accept it	.71	.36	
The threat is exaggerated by government/media		.68	
Medication will be available soon		.52	
It will not be as bad as predicted		.79	
It won't happen to me		.65	

Explained variance	22%	20%	17%

### Adaptive responses

**Figure 3:**
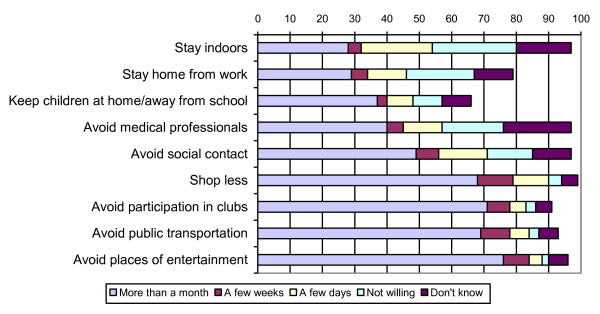
**Adaptive responses: For how long would you be willing to do this?**.

Avoiding places of entertainment, public transportation and clubs, as well as limiting shopping to the bare essentials were considered feasible protective measures by more than one third of the participants (see Figure 3). Approximately half of the sample was willing to avoid social contact if necessary and 40% reported willingness to avoid health care professionals. However, 21% were not sure how long they would be willing to avoid health care professionals. Of the 66% participants with children in school, 56% indicated a willingness to keep their children at home for longer than a month if necessary. Of the 77% participants that are employed, 38% indicated that they would be able to stay home from work more than a month and 27% stated that staying home would be impossible. Staying indoors was regarded as impossible by 26%, possible for a few days by 22%, and possible for longer than a month by 28%. An additional 17% indicated not knowing if they could take that measure.

### Communication and information

**Figure 4:**
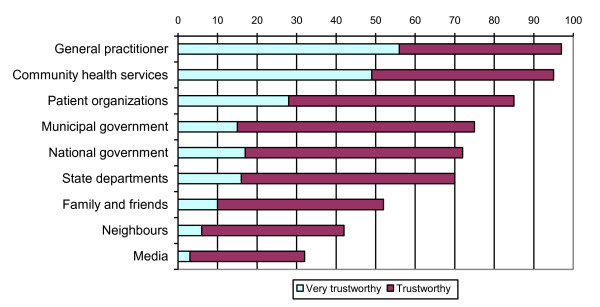
**Perceived trustworthiness of information sources**.

Regarding the perceived trustworthiness of information sources, participants reported thinking that their general practitioner and the community health services are most trustworthy (see Figure 4). Patient and consumer organizations were considered trustworthy by 85%. The municipal government, the national government and state departments were considered trustworthy by more than 70%. Family and friends, neighbours and the media were considered trustworthy but 52%, 42% and 32%, respectively.

**Figure 5:**
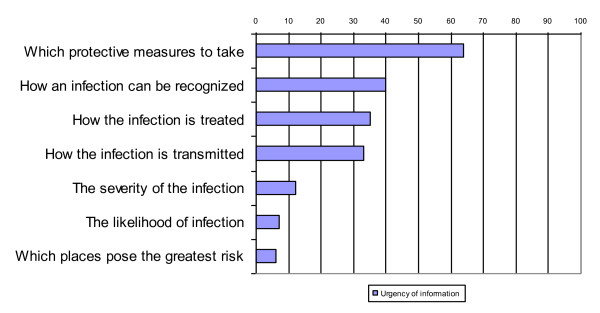
**Perceived urgency of information**.

Regarding the urgency of information, 64% reported wanting to receive information on protective measures immediately (see Figure 5). Information on how to recognize an infection was considered most urgent by 40% while information on how to treat the infection and how the infection is transmitted was considered most urgent by 35% and 33%, respectively. In general, topics that inform risk appraisal, namely information about the severity of the infection, the likelihood of infection and places that pose the greatest risk were considered less urgent.

### Scenarios

With respect to whether participants would be willing to stay home if they become ill, 61% said they certainly would and 34% stated that they probably would. Of these participants, 47% were certain that someone would take care of them and 38% thought that there would probably be someone to take care of them. In most cases, the potential caregiver was an immediate family member. With respect to caring for an ill family member, 62% reported being certain that they would be able to take care of their family member. An additional 32% claimed that they would probably be able to provide care. Among employed participants willing to provide care to an ill family member, the mean number of working hours per week was estimated to decline from 30.8 to 14.1.

In response to the scenario whereby participants were advised to stay home by the government but told to nonetheless come to work by their employer, 35% reported an intention to stay at home despite their employer's demands, 14% said they would work from home full time, 14% said they would work from home part time, 20% reported that they would simply go to work and 17% reported not knowing what they would do. Among the participants that reported an intention to stay home and who were subsequently provided with a scenario in which their choice would result in a loss of salary, the percentage of participants willing to stay home decreased from 35% to 20%. Participants were not inclined to believe that they could potentially lose some or all of their salaries if a pandemic were to occur. Only 5% of employed participants considered this a possibility, 17% thought it could happen but was unlikely, 62% found it pretty unlikely and 16% were convinced that such a situation would never occur. Despite perceptions that losing pay because of the pandemic was unlikely, 9% participants reported that not being paid would result in serious financial difficulties for their household within a week. An additional 42% claimed that this would generate problems after a month, 75% after a few months, 83% after half a year, 88% after a year and 96% after more than a year.

In response to the scenario in which children would not be able to go to school or child care facilities, 75% of all participants with children younger than 12 years (total *n *= 228) claimed that they would be able to care for their children themselves, 43% indicated that someone in their household would care for their children. In many cases, this person was their partner. Also, 24% reported that someone outside of their home would come and care for their children and 18% said that their children would be cared for by someone else at another location. Participants further indicated that, in order to care for children, a family member may need to stop working or, alternatively, work part time. Help from outside of the home was expected to be provided by family (77%), friends (9%) or neighbours (6%).

In response to a scenario whereby children would have to be isolated in the family home for three months, 24% of all participants with children younger than 17 (total *n *= 311) indicated that they would be successful in keeping their children at home and away from others, 37% said they would probably be successful, 27% claimed that they would probably not be successful and 6% indicated that they certainly would not be successful.

In response to the scenario pertaining to antiviral drugs, most participants indicated that they would certainly (39%) or probably (47%) want to use an antiviral drug. Also, 45% would certainly and 38% would probably be accepting of policy that prioritizes the treatment of vulnerable groups, such as the elderly. Even higher percentages were reported for the acceptance of policy that prioritizes professionals such as the police (62%) and fire fighters (32%). If availability of antiviral drugs were limited, 13% would certainly and 43% would probably attempt to obtain them through other means.

## Discussion

### Summary of the results

Knowledge levels regarding a possible influenza pandemic were mixed. Many participants reported not feeling well informed about protective measures. Further, the perceived susceptibility of an influenza pandemic was relatively low while perceived severity was quite high. Compared to a number of other diseases, risk perception was highest for an influenza pandemic Public transportation was considered the most risky place for acquiring an influenza infection. Further, most participants felt that there was little they could to prevent an infection.

Three types of maladaptive responses were distinguished: fatalism, underestimation and avoidance. Many participants felt that risks would be exaggerated by the government (underestimation), that a pandemic should simply be accepted as reality (fatalism) and that people are best off stocking up and staying indoors (avoidance). Low response and self efficacy may lead to fatalism, but high scores may lead to underestimation. Nevertheless, many participants reported a willingness to follow the recommendations of health professionals. Avoiding places of entertainment was considered easiest while staying indoors was thought to be the most difficult. Many people did not know how long they will be able to comply with that recommendation. General practitioners and local health authorities were considered the most trustworthy sources of information while the media was considered the least trustworthy. Trust in governmental bodies was relatively low. The information considered most urgent information pertained to protective measures.

If a situation were to arise in which family members need to be taken care of, working hours would likely decrease substantially. If a situation were to arise in which salaries cannot be paid, almost half of the participants would encounter serious financial problems after a month. If schools were to close, many parents would require help with child care arrangements and one parent would likely have to reduce his/her working hours. If available, many participants would be willing to take antiviral drugs. Most participants were accepting of policy that prioritizes vulnerable groups but nonetheless indicated that they would endeavour to obtain the drugs through other means.

### Results in the context of the current literature

Since our literature search in 2007, a number of studies that focus on risk perceptions and behavioural intentions related to an influenza pandemic have been published. Results of studies conducted prior to the current H1N1 Influenza pandemic show that, although there is support for proposed actions, people feel uncomfortable carrying out protective measures themselves [20-22]. Lau [23] found that a higher perception of risk was associated both with an influenza vaccination and with wearing a face mask. A Norwegian study showed that only 2% would not go to work if an influenza pandemic were occur [24] whereas, in China, much higher percentages were reported (17%) [25]. Rubin and colleagues reported on a study in the UK conducted in May 2009 during the current H1N1 influenza pandemic and showed that almost 40% of respondents reported having taken some kind of protective measure [26].

Our study corresponds with other studies that indicate that there is indeed a willingness to adopt recommended protective measures. However, in contrast to those studies, we have been able to gather more specific data on both maladaptive and adaptive behaviours as well as response tendencies in a number of possible scenarios.

### Limitations

All answers provided by the participants were hypothetical and indicative of intentions and expectations that may or may not translate into actual behaviours at the time of an influenza pandemic. Intentions do predict behaviours to a certain degree [27], but situational influences and unconscious or affective reactions are likely to have an independent effect. As such, additional research in controlled settings (i.e. behavioural laboratory) and in real life situations at the time of a new influenza outbreak is needed. The results of this study are derived from a cross-sectional analysis. In reality, changes in the development of the pandemic and the reactions of others may influence people's behavioural responses.

Most variables were measured at an overall level rather than with respect to specific behaviours. For future studies, it would be useful to measure determinants of behaviour specifically for each advised protective measure. Also, in addition to PMT variables, other more general theories may provide additional concepts that could enable a more extensive understanding of risky and protective behaviours. For example, the Theory of Planned Behaviour (TPB) [27] suggests that there may be beliefs connected to the risky behaviour other than risk or health beliefs. TPB also proposes the subjective social norm - other people's expectations - as a determinant of behaviour. Social Cognitive Theory (SCT) [28] has a strong focus on self efficacy and vicarious learning or modelling and thus would imply that the perceived behaviour of others should be considered. Self regulation theories [29] may help explain why people are successful or unsuccessful in changing their behaviour following a threat. Such theories suggest we explore people's awareness of the threat, their decision to act, goal setting and the planning of coping responses, implementation and relapse prevention. In a recent review on pandemic influenza risk perception, Leppin & Aro [30] also conclude that investing in more theory-based research is imperative.

## Conclusions and Implications

Maladaptive responses may cause people not to follow health authorities' recommendations for protective measures. Greater perceptions of risk may lead to fatalism and avoidance while lower perceptions of risk may cause underestimation. During a pandemic, messages from health authorities should anticipate this balance between overreacting and underreacting. It is clear that people will want to know what protective measures to take. Messages pertaining to protective measures are likely to be most effective if they originate from local (health) authorities and general practitioners as they are considered most trustworthy.

Nonpharmaceutical interventions may include the closure of schools. If that were to happen, many parents will be forced to stop working or to work less. If measures advised by the authorities are in conflict with the measures taken by companies, it is unclear what employees will do. Few people can cope with a loss of salary. In any case, high numbers of ill individuals will likely result in large numbers of people staying home and a decreased labour force in all sectors, including the health care sector.

## Competing interests

The authors declare that they have no competing interests.

## Authors' contributions

GK conceived the study, contributed to the interpretation of the data and drafted the manuscript. RJ and RG conducted the literature review, the survey and the analyses. RM, HS and OZ participated in the design of the study and the interpretation of the data. All authors read and approved the final text.

## Appendix

**Survey questions "Behavioral expectations regarding an influenza epidemic"**.

1. Are you familiar with the concept "influenza epidemic"?

◦ Yes, I know what it is

◦ I have heard of it but I am not sure what it is

◦ No, I have never heard of it

**A pandemic is a worldwide epidemic caused by a virus that is as yet unknown and for which no vaccine is available. An example is the Asian bird flu. When that virus infects people, a new and unknown form of influenza that is transmitted from person to person develops. It is not possible to predict whether or not a new influenza virus is in fact dangerous and will cause an epidemic or a pandemic**.

2. Were you familiar with the above information?

◦ Yes, completely

◦ Yes, mostly

◦ Yes, some of it

◦ Yes, vaguely

◦ No, not at all

**3. No one knows exactly how many people would get sick if an influenza pandemic were to occur. Please estimate how many people in the Netherlands you think would get sick if an influenza pandemic were to occur**.

___ % of all the people in the Netherlands would get sick

4. To what extent are you informed about the protective measures that could be taken if an influenza pandemic were to occur?

◦ Incredibly well informed

◦ Very well informed

◦ Well informed

◦ Reasonably informed

◦ Somewhat informed

◦ Poorly informed

◦ Very poorly informed

◦ I don't know

5. To what extent are you informed about the concepts influenza viruses and epidemics?

◦ Incredibly well informed

◦ Very well informed

◦ Well informed

◦ Reasonably informed

◦ Somewhat informed

◦ Poorly informed

◦ Very poorly informed

◦ I don't know

**6. A number of medical conditions are mentioned below. For each condition, please indicate how awful it would be if you were to be diagnosed with this condition in the coming 12 months. This can be done by selecting a number between 1 and 10. '1' means that it would not be awful at all and '10' means that it would be incredibly awful(see Table **3**).**

**Table 3:** Feelings on hypothetical diagnosis of listed medical conditions

		1	2	3	4	5	6	7	8	9	10	NA/I already have this condition	
HIV/AIDS		o	o	o	o	o	o	o	o	o	o	o	
Diabetes		o	o	o	o	o	o	o	o	o	o	o	
High blood pressure		o	o	o	o	o	o	o	o	o	o	o	
Tuberculosis		o	o	o	o	o	o	o	o	o	o	o	
A heart attack		o	o	o	o	o	o	o	o	o	o	o	
Food poisoning		o	o	o	o	o	o	o	o	o	o	o	
A common cold		o	o	o	o	o	o	o	o	o	o	o	
A common flu		o	o	o	o	o	o	o	o	o	o	o	
A flu from a new influenza virus		o	o	o	o	o	o	o	o	o	o	o	
A flu from a new influenza virus that has become a worldwide pandemic (also in the Netherlands)		o	o	o	o	o	o	o	o	o	o	o	

**7. How likely is it that you will be diagnosed with one of the following medical conditions in the next 12 months? '1' means not likely at all and '10' means very likely (see Table **4**).**

**Table 4:** Likelihood of diagnosis with listed medical conditions.

		1	2	3	4	5	6	7	8	9	10	NA/I already have this condition	
HIV/AIDS		o	o	o	o	o	o	o	o	o	o	o	
Diabetes		o	o	o	o	o	o	o	o	o	o	o	
High blood pressure		o	o	o	o	o	o	o	o	o	o	o	
Tuberculosis		o	o	o	o	o	o	o	o	o	o	o	
A heart attack		o	o	o	o	o	o	o	o	o	o	o	
Food poisoning		o	o	o	o	o	o	o	o	o	o	o	
A common cold		o	o	o	o	o	o	o	o	o	o	o	
A common flu		o	o	o	o	o	o	o	o	o	o	o	
A flu from a new influenza virus		o	o	o	o	o	o	o	o	o	o	o	
A flu from a new influenza virus that has become a worldwide pandemic (also in the Netherlands)		o	o	o	o	o	o	o	o	o	o	o	

8. Do you think that, in general, you are susceptible to getting the flu?

◦ Yes, very susceptible

◦ Yes, quite susceptible

◦ As susceptible as anyone else

◦ No, no really susceptible

◦ No, not susceptible at all

9. Imagine there is a worldwide influenza pandemic that has also reached the Netherlands. As a result, hospital admissions and deaths are on the rise. Where do you think you would be most likely to get infected?

◦ Public transportation

◦ Public places of entertainment like restaurants, bars and theaters

◦ Events like concerts and soccer games

◦ (Sport) clubs

◦ Shops and stores

◦ At work or in schools

◦ Hospitals

◦ At home or at the homes of friends and family

10. Are you scared that a worldwide influenza pandemic will occur?

◦ Constantly

◦ Frequently

◦ Sometimes

◦ Rarely

◦ Never

11. In general, do you think that people would be able take protective measures against a new influenza virus if a worldwide pandemic were to occur (also in the Netherlands)?

◦ I am very certain that measures could be taken

◦ I am quite certain that measures could be taken

◦ I am somewhat certain that measures could be taken

◦ I am doubtful that measures could be taken

◦ I am very certain that measures could NOT be taken

12. Do you think that YOU would be able to take protective measures against this new influenza virus if a worldwide pandemic were to occur (also in the Netherlands)?

◦ I am very certain that I could take the necessary measures

◦ I am quite certain that I could take the necessary measures

◦ I am somewhat certain that I could take the necessary measures

◦ I am doubtful that I could take the necessary measures

◦ I am very certain that I could NOT take the necessary measures

13. Imagine there is a new worldwide influenza pandemic that has also reached the Netherlands. Your local community health services have provided you with a face mask and urged you to wear it in all public places in order to prevent infection. Would you wear the face mask in all public places?

◦ Certainly

◦ Probably

◦ Probably not (skip ahead to question 15)

◦ Certainly not (skip ahead to question 15)

◦ I don't know (skip ahead to question 15)

14. If you wear a face mask, people may think that you have the new influenza and avoid you. The face mask also causes skin irritation. Would you still wear the face mask?

◦ Certainly

◦ Probably

◦ Probably not

◦ Certainly not

◦ I don't know

**15. Below are statements about what can happen if a new worldwide influenza pandemic were to occur (also in the Netherlands). Please indicate the degree to which you agree with each of these statements (see Table **5**).**

**Table 5:** Predicted consequences of a global pandemic.

**If a new worldwide influenza pandemic were to occur...**	Totally agree	Mostly agree	Don't agree or disagree	Totally disagree	Mostly disagree
I will move somewhere were there is no flu.	o	o	o	o	o
I will stock up and stay indoors	o	o	o	o	o
It will not be as bad as predicted	o	o	o	o	o
The threat will be exaggerated by the goverment and the media	o	o	o	o	o
We will all be completely powerless	o	o	o	o	o
There is nothing we can do about it	o	o	o	o	o
I will just have to accept it	o	o	o	o	o
Medication will quickly become available	o	o	o	o	o
It will not happen to me	o	o	o	o	o
I will be at an utter loss	o	o	o	o	o

**16. Imagine a new worldwide influenza pandemic occurs and this pandemic reaches the Netherlands. Within five weeks, 400,000 Dutch will become ill and 4,000 people will die. Health professionals recommend that you take the following protective measures indefinitely. How long would you be willing to take each of these measures (see Table **6**)?**

**Table 6:** Willingness to undertake preventative measures

	I am not willing	A few days	A few weeks	More than a month	I don't know	NA
Avoid public transportation	o	o	o	o	o	o
Avoid places of entertainment like restaurants, bars and theaters	o	o	o	o	o	o
Avoid (sport) clubs	o	o	o	o	o	o
Shop less and for necessary items only	o	o	o	o	o	o
Stay home from work	o	o	o	o	o	o
Keep kids at home and away from school	o	o	o	o	o	o
Avoid social contact with friends and family	o	o	o	o	o	o
Avoid medical professionals	o	o	o	o	o	o
Stay indoors	o	o	o	0	o	o

Before we continue with the survey, we would like to ask you a few questions about your current work situation.

17. Which of the following applies best to you right now?

◦ I am self-employed

◦ I have paid employment

◦ I work for the government

◦ I am disabled and thus unable to work

◦ I am unemployed/seeking employment/on welfare

◦ I am retired

◦ I study/go to school

◦ I am a stay-at-home mom or dad/housewife or househusband/other

18. On average, how many hours do you officially work per week? (This pertains to how many hours per normal/average work week are stipulated in your contract)

___ hours

◦ I do not work at this time

19. On average, how many of your contracted work hours do you work at home?

___ hours

◦ None/not applicable.

20. Including yourself, how many people are in your household?

___ people

21. Imagine there is a new worldwide influenza pandemic that has also reached the Netherlands. A lot of people fall ill. Only those that are very sick can be admitted to hospitals. The rest need to be cared for at home. You end up getting this new influenza virus and are advised by health professionals to stay home for 7 to 10 days and to have minimal contact with others. Would you be willing to do this?

◦ Certainly

◦ Probably

◦ Probably not (skip ahead to question 24)

◦ Certainly not (skip ahead to question 24)

◦ I don't know (skip ahead to question 24)

22. In this situation, would there be someone who would be able to take care of you (e.g. a family member, friend or neighbor)?

◦ Certainly

◦ Probably

◦ Probably not (skip ahead to question 24)

◦ Certainly not (skip ahead to question 24)

◦ I don't know (skip ahead to question 24)

23. Is the person that would likely care for you someone in your immediate family that lives with you or someone else?

◦ Immediate family

◦ Someone else

◦ Both

Questions 24 to 27 pertain only to people who live in households that comprise more than one person.

24. Imagine someone in your immediate family (a family member that lives in your household) becomes ill with the new influenza virus and health professionals have advised that you care for this person at home for 7 to 10 days. Would you be able to do this?

◦ Certainly

◦ Probably

◦ Probably not

◦ Certainly not

◦ I don't know

**Answer question 25 if question 24 was answered with a '1' or a '2' and if question 18 was answered affirmatively**.

25. How many hours would you still be able to work if you needed to care for this sick family member? 

___ Hours (max 2 numbers)

**Answer question 26 if question 24 was answered with '3' or '4' and if question 18 was answered affirmatively**.

26. Is there someone else in your household that would be able to care for this sick family member?

◦ Certainly

◦ Probably

◦ Probably not

◦ Certainly not

◦ I don't know

27. Imagine that, given the contagiousness and the severity of the new influenza virus, you would have to stay home for a month. Would you be able to work from home for a month?

◦ I would be able to do all my work at home

◦ I would be able to do some of my work at home

◦ I cannot work from home

**Answer questions 28 to 32 if question 17 was answered with '2' or '3'**.

28. Imagine there is a new worldwide influenza pandemic that has also reached the Netherlands. The government has said that all people that are infected need to stay home. You have symptoms of this flu but your employer says you must come to work. What would you do?

◦ Stay home and not work

◦ Work from home (skip ahead to question 30)

◦ Do some work from home (skip ahead to question 30)

◦ Just go to work (skip ahead to question 30)

◦ I don't know (skip ahead to question 30)

29. You would choose to stay home and not work. Imagine your employer says that while you are at home you will not be paid. What would you do?

◦ Still stay home and not work

◦ Work from home

◦ Do some work from home

◦ Just go to work

◦ I don't know

30. Imagine that health professionals advise businesses to close their doors. What do you think your company would do?

◦ Close its doors

◦ Stay open

◦ I don't know

31. Imagine that, because of the new influenza virus, you would be deprived of your salary (because you have to stay home or because your work has to close its doors). Can you imagine this actually happening?

◦ Yes, I can definitely imagine this happening

◦ Yes, I can imagine this happening but it seems unlikely

◦ No, this seems pretty unlikely

◦ No, this would never happen

32. If this were to happen (i.e. you would not be paid/receive salary), when would this result in serious financial problems?

◦ Within a week

◦ After a month

◦ After a few months

◦ After half a year

◦ After a year

◦ After more than a year

◦ Never

◦ I don't know/I would rather not say

Questions 33 to 39 pertain only to people who live in households that comprise more than one person.

33. Are there currently child(ren) under 12 years of age in your household?

◦ Yes

◦ No (skip ahead to question 38)

34. Imagine that all schools and child care services close for a month because of the new influenza virus. Who would take care of your child(ren) and where would this happen? (more than one answer is allowed)

▫ I would care for my child(ren) myself

▫ Someone else in my household would care for my child(ren)

▫ Someone outside my home would come and care for my child(ren) at my home

▫ Someone outside my home would care for my child(ren) at a location other than my home

▫ No one (all other options grey)

35. Would you or someone else in your household need to take time off of work in order to care for your child(ren)?

◦ Yes, I (or the other caregiver) would have to stop working.

◦ Yes, I (or the other caregiver) would have to work part time

◦ No

36. If all schools and child care services were to close, how much help from people outside your own household would need in order to keep your child(ren) at home for a month?

◦ A whole lot of help

◦ A fair amount of help

◦ Some help

◦ No help (skip ahead to question 38)

37. Outside of your own household, from whom would you expect to receive this help?

◦ My extended family

◦ My friends

◦ My neighbors

◦ My residents or neighborhood association

◦ The municipal government

◦ The community health services

◦ Another government agency

◦ My church or mosque

◦ Another club or organization

◦ Someone else

◦ I don't know/None of the above

38. Are there currently child(ren) under 17 years of age in your household?

◦ Yes

◦ No (skip ahead to question 40)

39. Imagine all schools and child care facilities close for three months in order to protect children from influenza infection. Health professionals have advised you to keep your child(ren) away from public places such as shopping centres and public transportation for three months. Children should also discontinue social contact with other children. Would you be able to keep your child(ren) from doing these things for three months?

◦ Certainly

◦ Probably

◦ Probably not

◦ Certainly not

◦ I don't know

**A vaccine for a new influenza virus can only be developed after the outbreak. There are, however, antiviral drugs such as Tamiflu and Relenza that may prevent the virus from dispersing through the body and that can shorten the illness period. However, it is unclear whether these antiviral drugs actually work for the new influenza virus**.

40. If a new influenza pandemic were to occur, would you want to use antiviral drugs?

◦ Certainly

◦ Probably

◦ Probably not

◦ Certainly not

◦ I don't know

41. Imagine there is a new worldwide influenza pandemic. The government has started distributing antiviral drugs to the population but there are not enough drugs for everyone. They have thus decided that those most vulnerable to infection (e.g. the elderly) should get priority access to the drugs. Do you think this is justified?

◦ Certainly

◦ Probably

◦ Probably not

◦ Certainly not

◦ I don't know

42. Also, certain professionals (e.g. firefighters and the police) are also more susceptible to infection. As a result, the government has decided to give these people priority access to antiviral drugs. Do you think this is justified?

◦ Certainly

◦ Probably

◦ Probably not

◦ Certainly not

◦ I don't know

43. These antiviral drugs are also being offered commercially on, for example, the internet. Imagine there is a new worldwide influenza pandemic and you cannot obtain antiviral drugs through your government because other groups are prioritized. Would you attempt to obtain these drugs through other means (i.e. buy them online)?

◦ Certainly

◦ Probably

◦ Probably not

◦ Certainly not

◦ I don't know

We now want to ask you a few questions about certain sources of information in the event of an influenza pandemic.

**44. In general, how trustworthy do you think the following sources of information are (see Table **7**)?**

**Table 7:** Perception of trustworthiness of information sources.

	Not Very trustworthy	Reasonably trustworthy	Not very trustworthy	trustworthy at all	I don't know
The government in general	o	o	o	o	o
The current national government	o	o	o	o	o
State departments	o	o	o	o	o
The municipal goverment	o	o	o	o	o
The community health services	o	o	o	o	o
The European Union	o	o	o	o	o
General practitioners	o	o	o	o	o
The media	o	o	o	o	o
Corporate enterprises	o	o	o	o	o
Consumer or patient organizations	o	o	o	o	o

**45. If a new worldwide influenza pandemic were to occur, information could be provided to you by the following sources. How trustworthy would you consider the information from these sources to be (see Table **8**)?**

**Table 8:** Perception of trustworthiness of information sources in the event of a global pandemic.

	Not Very trustworthy	Reasonably trustworthy	Not very trustworthy	trustworthy at all	I don't know
The current national government	o	o	o	o	o
State departments	o	o	o	o	o
The municipal goverment	o	o	o	o	o
The community health services	o	o	o	o	o
The European Union	o	o	o	o	o
My general practitioner	o	o	o	o	o
The media	o	o	o	o	o
Consumer or patient organizations	o	o	o	o	o
My family and/or friends	o	o	o	o	o
My neighbors	o	o	o	o	o

46. Imagine a new worldwide influenza pandemic occurs and that the pandemic reaches the Netherlands. Which two topics would you want to receive immediate information about?

▫ How the infection is transmitted

▫ How the infection can be recognized

▫ Which protective measures you can take to protect yourself against infection

▫ Which places pose the greatest risk

▫ The likelihood of infection

▫ How the infection is treated

▫ The severity of the infection

This is the end of the survey. Thank you for cooperating. If you have questions or comments, feel free to use the space below.

Please click on 'Next' to submit the survey.

## Pre-publication history

The pre-publication history for this paper can be accessed here:

http://www.biomedcentral.com/1471-2458/10/174/prepub

## References

[B1] WHO Pandemic preparedness Retrieved May 26th 2009 http://www.who.int/csr/disease/influenza/pandemic/en/index.html

[B2] Brewer NT Chapman GB Gibbons FX Gerrard M McCaul KD Weinstein ND Meta-analysis of the relationship between risk perception and health behaviour: The example of vaccination Health Psychology 2007 26 136 145 10.1037/0278-6133.26.2.136 17385964

[B3] De Zwart O Veldhuijzen IK Elam G Aro AR Abraham T Bishop GD Voeten HA Richardus JH Brug J Perceived threat, risk perception, and efficacy beliefs related to SARS and other (emerging) infectious diseases: results of an international survey International Journal of Behavioural Medicine 2009 16 30 40 10.1007/s12529-008-9008-2 PMC2691522 19125335

[B4] Sadique MZ Edmunds WJ Smith RD Meerding WJ de Zwart O Brug J Beutels P Precautionary behaviour in response to perceived threat of pandemic influenza Emerging Infectious Diseases 2007 http://www.cdc.gov/EID/content/13/9/1307.htm 18252100 10.3201/eid1309.070372 PMC2857294

[B5] Siu W Extended Parallel Process Model and H5N1 influenza virus Psychological Reports 2008 102 539 550 10.2466/PR0.102.2.539-550 18567223

[B6] Norman P Boer H Seydel ER Conner M, Norman P Protection Motivation Theory Predicting health behaviour 2005 Berkshire, UK: Open University Press 81 126

[B7] Champion VL Skinner CS Glanz K, Rimer BK, Viswanath K The Health Belief Model Health behaviour and health education; theory, research, and practice 2008 San Francisco, CA: Jossey Bass 45 65

[B8] Witte K Andersen PA, Guerrero LK Fear as motivator, fear as inhibitor: Using the EPPM to explain fear appeal successes and failures The handbook of communication and emotion 1998 New York: Academic Press 423 450

[B9] Weinstein ND Sandman PM Blalock SJ Glanz K, Rimer BK, Viswanath K The Precaution Adoption Process Model Health behaviour and health education; theory, research, and practice 2008 San Francisco, CA: Jossey Bass 123 147

[B10] De Hoog N Stroebe W de Wit JBF The impact of vulnerability to and severity of a health risk on processing and acceptance of fear-arousing communications: A meta-analysis Review of General Psychology 2007 11 258 285 10.1037/1089-2680.11.3.258

[B11] Ruiter RAC Abraham C Kok G Scary warnings and rational precautions: A review of the psychology of fear appeals Psychology & Health 2001 16 613 630 10.1080/08870440108405863

[B12] Markel H Lipman HB Navarro JA Sloan A Michalsen JR Stren AM Cetron MS Nonpharmaceutical interventions implemented by US cities during the 1918-1919 influenza pandemic JAMA 2007 298 644 654 10.1001/jama.298.6.644 17684187

[B13] Hong S Collins A Societal responses to familiar versus unfamiliar risk: Comparisons of influenza and SARS in Korea Risk Analysis 2006 26 1247 1257 10.1111/j.1539-6924.2006.00812.x 17054529

[B14] Voeten HA de Zwart O Veldhuijzen IK Yuen C Jiang X Elam G Abraham T Brug J Sources of information and health beliefs related to SARS and avian influenza among Chinese communities in the United Kingdom and The Netherlands, compared to the general population in these countries International Journal of Behavioural Medicine 2009 16 49 57 10.1007/s12529-008-9006-4 PMC7090907 19184453

[B15] Vartti AM Oenema A Schreck M Uutela A de Zwart O Brug J Aro AR SARS knowledge, perceptions, and behaviours: a comparison between Finns and the Dutch during the SARS outbreak in 2003 International Journal of Behavioural Medicine 2009 16 41 48 10.1007/s12529-008-9004-6 PMC7091200 19184625

[B16] Fishhoff B Gonzales RM Small DA Lerner JS Evaluating the success of terror risk communication Biosecurity and Bioterrorism: Biodefense Strategy, Practice, and Science 2003 1 255 258 10.1089/153871303771861450 15040205

[B17] Crimando S Accurate disaster behavioural response planning; a guide for business continuity planners 2006 New York: Extreme Behavioural Risk Management LLC http://www.xbrm.com/

[B18] Rippetoe PA Rogers RW Effects of components of Protection-Motivation Theory on adaptive and maladaptive coping with a health threat Journal of Personality and Social Psychology 1987 52 596 604 10.1037/0022-3514.52.3.596 3572727

[B19] Vlek C Stallen PJ Rational and personal aspects of risk Acta Psychologica 1980 45 273 300 10.1016/0001-6918(80)90038-4

[B20] Paek HJ Hilyard K Freimuth VS Barge JK Mindlin M Public support for government actions during a flu pandemic: lessons learned from a statewide survey Health Promotion Practice 2008 9 60S 72S 10.1177/1524839908322114 18936261

[B21] Raude J Setbon M Lay perceptions of the pandemic influenza threat European Journal of Epidemiology 2009 24 339 42 10.1007/s10654-009-9351-x 19484363

[B22] Lau JT Kim JH Tsui HY Griffiths S Perceptions related to bird-to-human avian influenza, influenza vaccination, and use of face mask Infection 2008 36 434 43 10.1007/s15010-008-7277-y 18795229 PMC7099207

[B23] Smith BW Kay VS Hoyt TV Bernard ML Predicting the anticipated emotional and behavioural responses to an avian flu outbreak American Journal of Infection Control 2009 37 371 80 10.1016/j.ajic.2008.08.007 19121548 PMC7124230

[B24] Kristiansen IS Halvorsen PA Gyrd-Hansen D Influenza pandemic: perception of risk and individual precautions in a general population. Cross sectional study BMC Public Health 2007 7 48 10.1186/1471-2458-7-48 17407563 PMC1852795

[B25] Yuan J Zhang L Xu W Shen J Zhang P Ma H Reported changes in health-related behaviours in Chinese urban residents in response to an influenza pandemic Epidemiology and Infection 2009 137 988 993 10.1017/S0950268809002726 19426571

[B26] Rubin GJ Amlôt R Page L Wessely S Public perceptions, anxiety, and behaviour change in relation to the swine flu outbreak: cross sectional telephone survey BMJ 2009 339 b2651 10.1136/bmj.b2651 19574308 PMC2714687

[B27] Montaño DE Kasprzyk D Glanz K, Rimer BK, Viswanath K Theory of Reasoned Action, Theory of Planned Behaviour and the integrated model Health behaviour and health education; theory, research, and practice 2007 San Francisco, CA: Jossey Bass 67 96

[B28] McAlister AL Perry CL Parcel GS Glanz K, Rimer BK, Viswanath K How individuals, environments, and health behaviours interact: Social Cognitive Theory Health behaviour and health education; theory, research, and practice 2007 San Francisco, CA: Jossey Bass 169 188

[B29] Zimmerman BJ Boekaerts M, Pintrich PR, Zeidner M Attaining self-regulation Handbook of self-regulation 2000 London: Academic press 13 39 full_text

[B30] Leppin A Aro AR Risk perceptions related to SARS and avian influenza: theoretical foundations of current empirical research International Journal of Behavioural Medicine 2009 16 7 29 10.1007/s12529-008-9002-8 PMC7090865 19214752

